# Insights into the localized corrosion initiation mechanisms of alloy 718

**DOI:** 10.1016/j.heliyon.2025.e41754

**Published:** 2025-01-08

**Authors:** Julia Botinha, Bodo Gehrmann, Helena Alves, Michael Rohwerder

**Affiliations:** a VDM Metals International GmbH, Kleffstraße 23, 58762, Altena, Germany; b Max Planck Institute for Sustainable Materials, Max-Planck-Straße 1, 40237, Düsseldorf, Germany

**Keywords:** Nickel based-alloy, Polarization, SEM, Inclusion, Interfaces, Pitting corrosion

## Abstract

Nickel alloys are widely used in the oil and gas industry where high corrosion resistance in chloride water or in sour environments is required. When high mechanical properties are required in combination with high corrosion properties, alloy 718 (UNS N07718) is one of the preferred choices, although it still presents limitations in terms of corrosion resistance in some applications: despite offering outstanding resistance to localized corrosion, alloy 718 is not immune to it. Its high corrosion resistance is mainly due to its high nickel and chromium contents combined with additions of molybdenum and other noble elements. Metallic and non-metallic precipitates are formed during the manufacturing process and each is known to interact with the matrix and the oxide protective film, but there is a lack of studies on the mechanisms that occur during the initiation of localized corrosion in this alloy. In this work, we investigate the localized corrosion initiation process and follow up the first steps of corrosion attack in alloy 718 in a 1M NaCl solution through the combination of microscopy and electrochemical techniques. As a result of potential application in a brine electrolyte, we conclude that nitrides and carbides play an important role on the corrosion initiation.

## Highlights

• Precipitates are responsible for localized corrosion initiation in alloy 718.
• Alloy 718's main precipitation types are titanium nitrides and niobium carbides.
• Nano-crevices inside precipitates induce corrosion initiation on alloy's 718 matrix.

## 1 Introduction

Consisting primarily of nickel, chromium, iron, molybdenum, niobium, titanium and aluminium, alloy 718 has been developed in the early 1960s by H. L. Eiselstein [1] for the aerospace industry and it is until today one of the mostly utilized nickel alloys in the oil and gas industry. Along the years and with the industrial technological advances, the applications requirements have increased, what led to stressing the materials further and further to their limits, while keeping a conservative material selection by relying on historical alloys instead of selecting new ones.

Alloy 718 finds extensive use in various components such as completion tools, packers, hangers, valves, flanges, and downhole measurement tools [[2], [3], [4], [5], [6]]. These applications require materials with high strength, toughness, pitting and general corrosion resistance, hydrogen embrittlement resistance and stress corrosion cracking resistance. The combination of properties presented by Alloy 718 that allow such extensive application is a result of the alloy's complex microstructure, which is an outcome of its heat treatment condition, which is constituted by a solution annealing followed by age hardening [7].

By carrying out a first heat treatment step at a temperature above the delta phase dissolution, which is high enough to dissolve the intermetallic phases that might occur in alloy 718, an austenitic face-centered-cubic gamma matrix predominantly composed by nickel, chromium and iron is created [8,9], promoting a homogeneous grain size distribution through grain recrystallization [[10], [11], [12]]. The substantial presence of nickel and chromium in the matrix enables excellent resistance to general and cracking corrosion, while iron is incorporated to manage the metal cost. According to H. L. Eiselstein [13], a nickel content above 50 % is required by the alloy design to guarantee maximum yield strength, considering that enough nickel must be available for the later formation of intermetallic phases that are responsible for the hardening effect. Molybdenum is added to the alloy at approximately 3 wt.-% and, together with the chromium in solid solution within the gamma phase that composes the matrix of alloy 718, provide acceptable resistance against localized corrosion in chloride-containing environments. These elements are responsible for the passive and repassivation behaviours in nickel-based alloys, acting directly in the passive film formation, which is mainly composed by chromium oxides, and reducing the passive current of the alloy, thus reducing the rate of acidification in the pit environment due to the formation of molybdenum oxides [14,15]. Niobium, titanium, and aluminium, along with a portion of nickel, play a crucial role in precipitating intermetallic phases such as gamma prime, gamma double prime and delta [2]. Gamma prime is a cubic face-centered L1_2_ ordered Ni_3_(Al,Ti) phase and the gamma double prime is the metastable phase (Ni_3_Nb), with tetragonal space centered crystal structure DO22 [16]. Both gamma prime and gamma double prime phases are precipitated during the age hardening heat treatment and significantly contribute to the improvement of mechanical properties in alloy 718, through dislocation-particle interaction, where the dislocations bypass the particles either by Orowan looping or cross slip [17]. When exposed to temperature for sufficient time, gamma double prime transforms into the stable delta phase, with orthorhombic DO_a_-ordered crystal structure [9,18]. However, the delta phase is undesirable in oil and gas applications due to its negative impact on the corrosion and cracking properties of the alloy, as elucidated by several authors [6,19,20]. Luo et al. [21] shows that the incoherent interface between the needle-like delta phase and the gamma matrix produces a serious lattice misfit, accelerating anodic dissolution and consequently leading to an easier breakdown of surface passive film in the vicinity of phase boundaries. Those observations can be amended by Mannan et al. [22], who add that this corrosion initiation close to the phase interfaces with the matrix can even lead to increased stress corrosion cracking susceptibility.

Despite the overall favourable properties of alloy 718, it remains susceptible to localized corrosion under specific conditions and, as a result, to environmentally assisted cracking, even when the manufacturing process is designed to provide a microstructure that is free from the undesired delta phase.

Being enriched in chromium and molybdenum, alloy 718 takes advantage from corrosion protection by passivity [15,23,24]. Different models for passivity breakdown exist [[24], [25], [26], [27], [28], [29], [30], [31], [32]] and it is known that localized corrosion, once initiated, propagates due to the extreme acidified condition that is electrochemically developed due to metal ion hydrolysis and maintained within a localized zone [33].

As elucidated before, the negative effects of delta phase precipitation in the grain boundaries of alloy 718 are well known. However, if manufacturing process enables the production of the alloy without or even with very low amounts of delta phase, the mechanism for pitting initiation is still not deeply understood.

During the manufacturing process and due to the affinity of carbon and nitrogen with the alloying elements, carbides, nitrides and carbonitrides are formed and present in the alloy. These are formed either during the solidification process or during the annealing heat treatment [9] and are identified to be of the MC(N) type presenting a cubic face-centered crystal structure [16,18,34]. These phases are considered to be stable and will not change with thermal cycles at lower temperatures, e.g. during age hardening heat treatments [35].

Klöwer et al. [36] conducted an extensive research program to investigate the effects of precipitates on the susceptibility of alloy 718 to pitting corrosion, which serves as a precursor to environmentally assisted cracking mechanisms. Their findings indicated that the delta phase, gamma prime, and gamma double prime phases have no significant effect on the potential and nucleation time of pits in the studied chloride-containing environments. Instead, they identified carbides and carbonitrides on the material's surface acting as preferential sites for pitting initiation, although a mechanistic analysis has not been conducted.

Garfias-Mesias et al. [37] employed in-situ microscopy to identify the precursor sites for pitting initiation in alloy 718 exposed to an acidified chloride-containing solution at increasing temperatures. The authors concluded that stable pits originated from particles rich in niobium and carbon, but were not able to conduct any mechanistic approach as well.

E. Alekseeva et al. [38] utilized electrolytic extraction techniques and scanning electron microscopy to assess the impact of inclusions on the corrosion resistance of alloy 718 by examining the dissolution extent of the matrix surrounding the inclusions. They identified three typical types of inclusions in alloy 718: NbTi-C carbides, TiNb-N nitrides, and NbTiCr-C carbides. The authors concluded that the position of the inclusions (whether at grain boundaries or within grains) and their composition affect the corrosion resistance of the alloy. According to their findings, nitrides and small carbides located at grain boundaries are the most detrimental inclusions to the corrosion resistance of alloy 718.

Similarly, D. Gisinger et al. [39] investigated the pitting corrosion resistance of alloy 718 in different NaCl-containing solutions using electrochemical and scanning electron microscopy methods. They also observed that the interface between Nb-rich particles and the matrix serves as a preferential site for pit initiation.

In general, it is proposed in the literature that local depassivation and pitting initiation can be promoted by the presence of microstructural defects of the metal substrate. The oxide layer formed above interfaces and defects of the metal substrate contain potentially less resistive defective regions that are more sensitive to breakdown [[25], [26], [27]]. Islam et al. [40] modelled the interface between the metal and its oxide layer and gave focus on the microvoid formation in these interfaces, which challenge adherence and lead to an earlier breakdown of the protective layer.

In a general discussion about localized corrosion, R. Newman [41] points out that pit initiation occurs within a micro-crevice and/or an impurity particle and questions if initiation would be possible without these, but literature data and further discussion is not available.

Despite the work available in the prevailing literary repertoire, a comprehensive understanding of the influence of microstructural characteristics and their interactions with respect to corrosion resistance has not been fully achieved. The aim of this work is to add value to the existing body of literature by investigating the pitting initiation process and following up the first steps of corrosion attack in Alloy 718, as valuable information for a later understanding of possible mechanisms that might be occurring. Knowing from the current state of the art that carbides, nitrides and carbonitrides are frequently correlated to pitting initiation in Alloy 718, a better understanding of the mechanisms taking place is of great importance as a tool to mitigate its occurrence and possible field failures.

## 2 Material and methods

Commercially available alloy 718 with chemical composition according to the UNS number N07718 has been used to carry out the investigations. The main chemical composition of the alloy used is shown on Table 1. Additional impurity elements are present.

**Table 1:** Main typical chemical composition of alloy 718 in weight percent.

Ni	Cr	Fe	Mo	Nb + Ta	Ti	Al	C	P
53.0	18.5	Balance	3.00	5.00	1.00	0.50	0.02	0.003

**Fig. 1:**
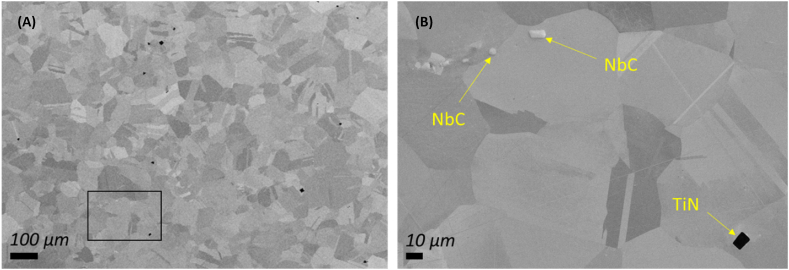
SEM metallography of alloy 718 showing recrystallized austenite grains and dispersed NbC and TiN precipitates at (A) 100X and (B) 1200× magnification. The region delimited on (A) is the zoomed area shown on (B).

**Fig. 2:**
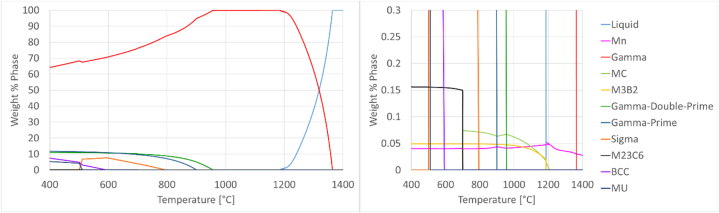
Thermodynamically calculated weight fraction of the different phases present in alloy 718. The calculations were done using the software JMatPro. Weight percentages from 0.0 to 0.3 % have been zoomed on the diagram on the right side to depict the contents and precipitation temperatures of Nitrides and Carbides, which are present in very low amounts.

The alloy has been submitted to heat treatment as required by the main specification that norms the products used in the oil and gas industry, the API 6ACRA [7]. This consists of a solution anneal at a temperature between 1021 and 1052 °C for 1–3 h, followed by an age hardening at a temperature between 774 and 802 °C for 6–8 h. This heat treatment schedule produces a homogeneous microstructure as shown on Fig. 1, composed by fully recrystallized austenite grains with a grain size of 108 μm, determined according to ASTM E112 [42] using the intercept procedure. A more detailed view of the delimited area from Fig. 1 (A) is given on (B). Carbides and nitrides with dimensions in the μm-range are visibly dispersed in the gamma matrix, as in agreement with the thermodynamically calculated weight fractions of the different phases present in alloy 718 that is shown on Fig. 2. The calculations were done using the multi-platform software program JMatpro, an acronym for Java-based materials properties. Nitrides and carbides are present in quantities below 0.1 wt.-% at temperatures corresponding to the heat treatment cycles mentioned above. The intermetallic phases gamma prime and gamma double prime are in the nanometer size range and therefore cannot be detected through the employed metallographic methodology.

**Fig. 3:**
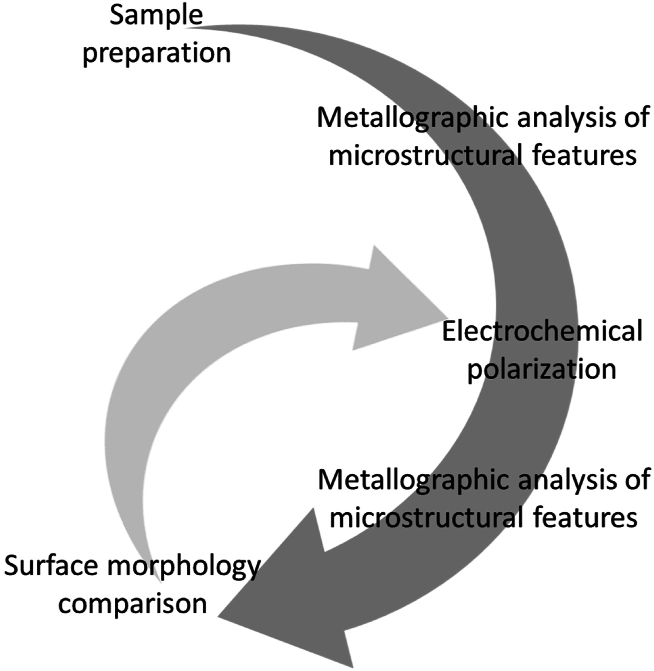
Systematic protocol of sample investigation.

In pursuit of establishing a correlation between corrosion initiation sites and material defects or other metallographic features, a systematic protocol, as depicted in Fig. 3, has been devised and diligently executed for each experimental trial. Initially, the specimens are prepared, and the ensuing surface characteristics are meticulously recorded. Subsequently, the sample undergoes electrochemical polarization. After the polarization process, the surface of the specimen is once again comprehensively investigated, and the acquired metallographic data are scrutinized by means of a comparative analysis between the pre-polarization and post-polarization surface features. Thereafter, the sample is polarized again. This procedure is repeated several times.

For the sample preparation, cubic samples having about 1x1x1 mm first undergo passivation in a 20 % HNO_3_ solution for a duration of 60 min to establish a stable passive layer on their surfaces that later will be in contact with the mounting material. This process aims to mitigate potential preferential corrosion attack at those contact boundaries. Subsequent to passivation, the samples are embedded in the PolyFast conductive resin from Struers using the Struers CitoPress-15 mounting equipment for hot mounting to allow their examination under scanning electron microscopy.

Following the mounting process, the samples undergo grinding to achieve a P2500 surface finish. Subsequently, they are polished successively using diamond suspensions with particle size of 3 μm and 1 μm, respectively, finishing with oxide polishing suspension (OPS) for a better surface quality.

Following the completion of the sample polishing process, the polished surfaces are characterized employing a Zeiss Leo Gemini 1550VP Scanning Electron Microscope (SEM) equipped with a Secondary Electron (SE), an Energy-Dispersive X-Ray (EDX) detector and a Backscattered electron (BSE) detector from EDAX. The stage-scan function is utilized to automatically record specific regions of interest within the stage where the sample is placed. This process involves dividing the designated sample area into partial images with controlled overlap based on the predetermined magnification setting. Subsequently, these individual images are assembled to reconstruct the complete image using the Fiji ImageJ [43] software, which is readily available for public access.

In addition to the SEM image examination of the total sample surface area, specific inclusions and precipitates visible at a magnification of 3500× are selectively (in a random manner) subjected to analysis through Energy Dispersive X-ray Spectroscopy (EDS). The objective is to establish a comprehensive database of particles present in the material, and, subsequently, correlate them with their corresponding shapes and grey scale characteristics. The decision to utilize a magnification of 3500× is based on preliminary investigations carried out by the authors that have indicated localized corrosion typically initiates in particles ranging from 1 to 100 μm in size.

**Fig. 4:**
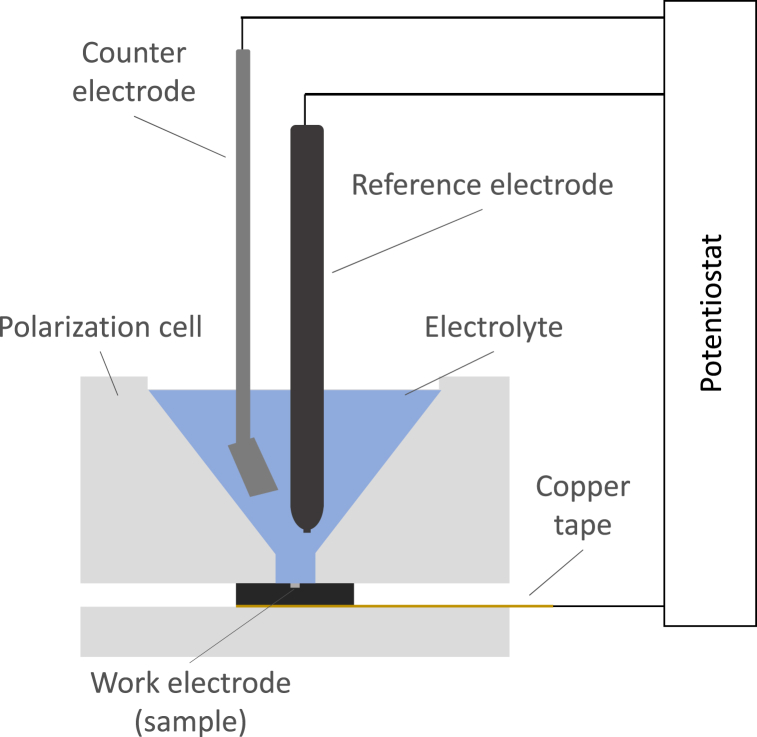
Schematic representation of electrochemical cell.

After surface characterization, the sample is subjected to a polarization cycle. Fig. 4 illustrates the schematic representation of the electrochemical cell employed in the study. A 1M NaCl solution serves as the electrolyte across all experimental trials. The reference electrode is Ag/AgCl, while the counter electrode consists of a slim platinum strip with sufficient surface area to accommodate the current generated by the working electrode. Circuit closure is achieved using a PalmSens EmStat3+ potentiostat, enabling the imposition of a constant potential between the reference and working electrodes for a specified duration. During this period, the corresponding current is monitored. Following the completion of the potentiostatic application, the circuit is automatically open and the measurement is finished.

Subsequent to the potentiostatic polarization, the sample surface is subjected to SEM imaging, mirroring the observation and analysis process prior to polarization. When the post-polarization images are available, a comparative analysis is conducted between the sample surfaces before and after the polarization event. Discrepancies observed in the samples before and after polarization are meticulously recorded and documented.

Based on the outcomes and the presence or absence of corrosion manifestations on the surface, the option to subject the sample to additional electrochemical re-polarization at a higher potential and/or for longer durations is considered.

In order to avoid indiscriminate and fast corrosion rates, which would prevent the observation of initial corrosion steps, operational parameters are established by consecutively executing the outlined procedural steps while systematically adjusting the applied potentials by increasing it stepwise. This process continues until localized corrosion is discernible in the samples, occurring at 1050 mV vs. the Ag/AgCl reference. Being the first potential at which samples showed slightly corrosion attack, the potential of 1050 mV, maintained for a duration of 3 min, is designated as the initiation point for this investigative endeavor. Subsequently, re-polarization sequences are executed successively for durations of 5, 10, 20, 30, and 60 min.

## 3 Results and discussion

**Fig. 5:**
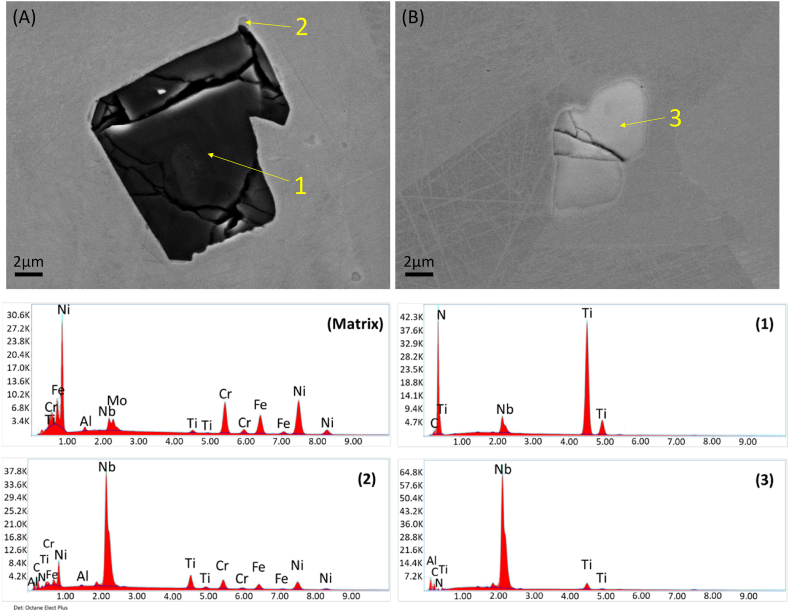
SEM micrographs of (A) dark-grey and (B) light-grey particles in alloy 718. EDX spectrums taken from the matrix and from the spots indicated with an arrow numbered from 1 to 3 are shown. Dark-grey particles are mainly composed by titanium and nitrogen whereas light-grey particles are mainly composed by niobium and carbon.

**Fig. 6:**
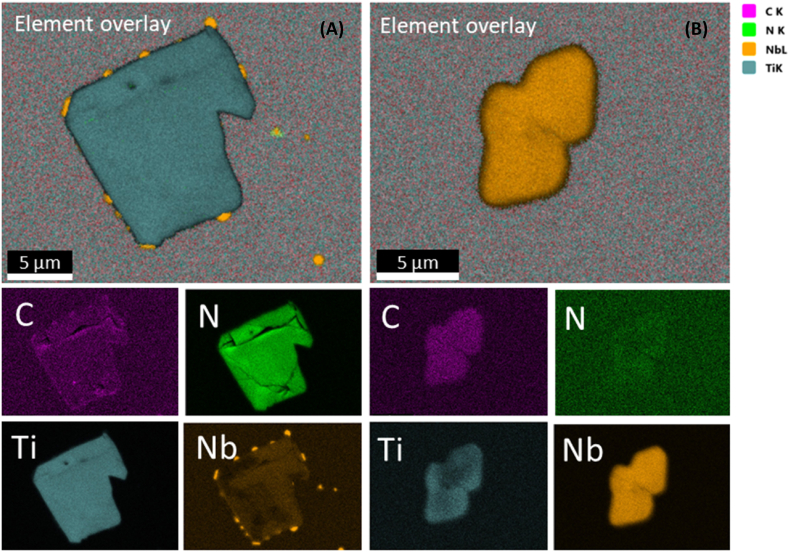
EDS mapping of “dark-grey” and “light-grey” particles in alloy 718. (A) “dark-grey” particle containing mostly nitrogen and Titanium. (B) “light grey” particle containing mostly Niobium and Carbon.

Upon a comprehensive examination of multiple samples, we draw inference to the existence of two main distinct particle categories that potentially contribute to the initiation of localized corrosion phenomena. These particles have been classified into “dark-grey” and “light-grey” particles, predicated on their discernible contrast with the matrix in the SEM micrographs, thoughtfully represented in Fig. 5. Identification numbers are assigned to the locations marked by yellow arrows, which signify the regions designated for EDS analysis, for which EDS spectra were captured using an Octane Elect Plus detector from EDAX. The fitted main atomic and weight compositions characterizing representative instances of “dark-grey” and “light-grey” particles, as well as the matrix are given on Table 2. The “dark-grey” particles predominantly exhibit a chemical composition characterized by an abundance of nitrogen and titanium. Conversely, the “light-grey” particles are niobium-rich, primarily constituted of carbon. A mapping analysis is additionally presented in Fig. 6. Remarkably, the co-occurrence of “dark-grey” and “light-grey” particles is frequently observed, as exemplified in Fig. 5, Fig. 6, where dark grey particles seems to act as a nucleation site for smaller light-grey ones.

**Table 2:** Fitted atomic- and weight-chemical composition of matrix and spots 1 to 3.

Spot	C	N	Al	Ti	Cr	Fe	Ni	Nb	Mo
**Atomic-%**									
Matrix			1.6	1.2	20.1	16.9	55.2	3.1	1.8
1	7.5	57.3	0.1	32.9	0.2		0.2	1.8	
2	61.7	4.6	0.3	0.6	4.2	3.3	8.2	17.1	
3	67.0	8.5	0.2	2.7	1.1		0.5	19.8	
**Weight-%**									
Matrix			0.8	1.0	18.0	16.3	56.0	4.9	3.0
1	3.4	30.2	0.1	59.1	0.4		0.5	6.3	
2	22.4	2.0	0.2	0.8	6.6	5.5	14.6	47.9	
3	26.9	4.0	0.2	4.4	2.0		1.1	61.4

The two selected particles that are shown on Fig. 5, Fig. 6 and Table 2 are representative of similar particles and their chemical compositions lie within the common composition range for these particle types, where “dark-grey” and “light-grey” refers to their contrast with the matrix.

**Fig. 7:**
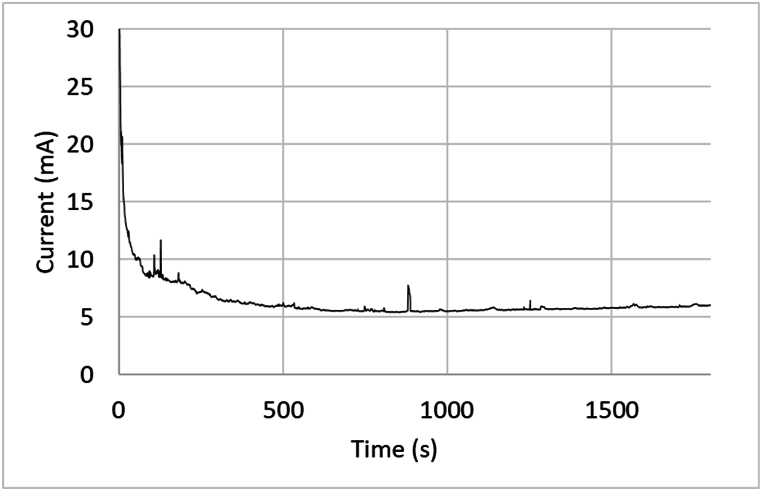
Current dependence on polarization time at a potential of 1050 mV in 1M NaCl electrolyte. The small current peaks are indicative of metastable pitting events.

Following each polarization cycle, the specimens underwent analysis through scanning electron microscopy (SEM) utilizing the stage scan function described above. The dependence of the current on the polarization time is illustrated in Fig. 7, where the sample was polarized for a duration of 30 min. This graph is shown for illustration purposes, although different (shorter and longer) polarization times have been applied in this study. A consistent pattern emerged across the majority of the polarized samples, characterized by small current peaks indicative of metastable pitting events. These events are subsequently followed by repassivation process, ultimately restoring the current to its pre-event stability level.

**Fig. 8:**
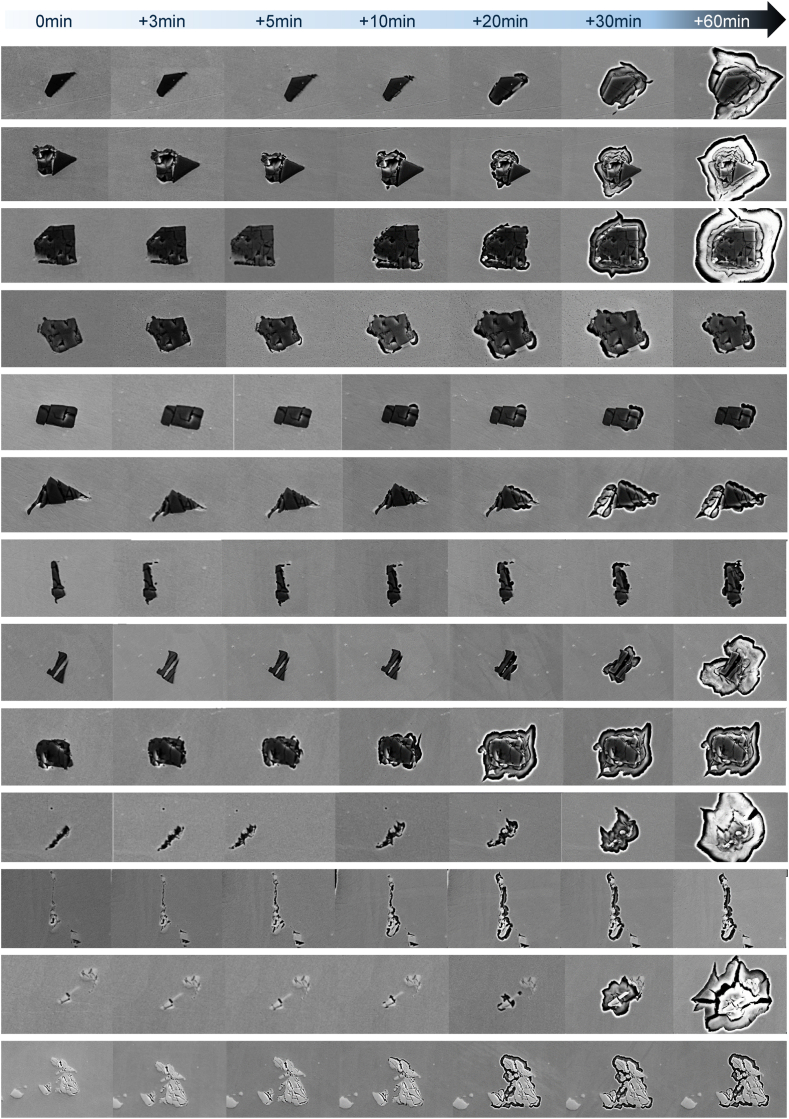
Evolution of corrosion attack close to particles after subsequent polarizations. Corrosion starts on the matrix, on its boundaries with both titanium nitrides (dark grey particles) or niobium carbides (light grey particles) and continues evolving with further potential application, until, in most of the cases, the surrounding of the particle is completely corroded.

Fig. 8**Fehler! Verweisquelle konnte nicht gefunden werden.** elucidates the evolution of corrosion attack at the boundary between particle and matrix featuring the particle types that were discussed above. The time scale above the metallographic images show the duration of potential application at 1050 mV, where “0min” indicate the initial condition of particles that showed corrosion attack in their vicinities in the next polarization cycles. As the total polarization time increases, the localized corrosion attack gradually develops in the matrix, in its boundaries with the affected particles. These observations go well with the literature evidences available [[36], [37], [38], [39]] that carbides, carbonitrides and nitrides act as preferential sites for localized corrosion. While Fig. 8 visually presents a higher incidence of corrosion attack on the boundaries of the matrix with “dark-grey” particles, establishing a direct correlation between corrosion frequency and particle type remains elusive. Nonetheless, a discernible feature, presumably associated with a geometrical factor, has emerged with our results.

**Fig. 9:**
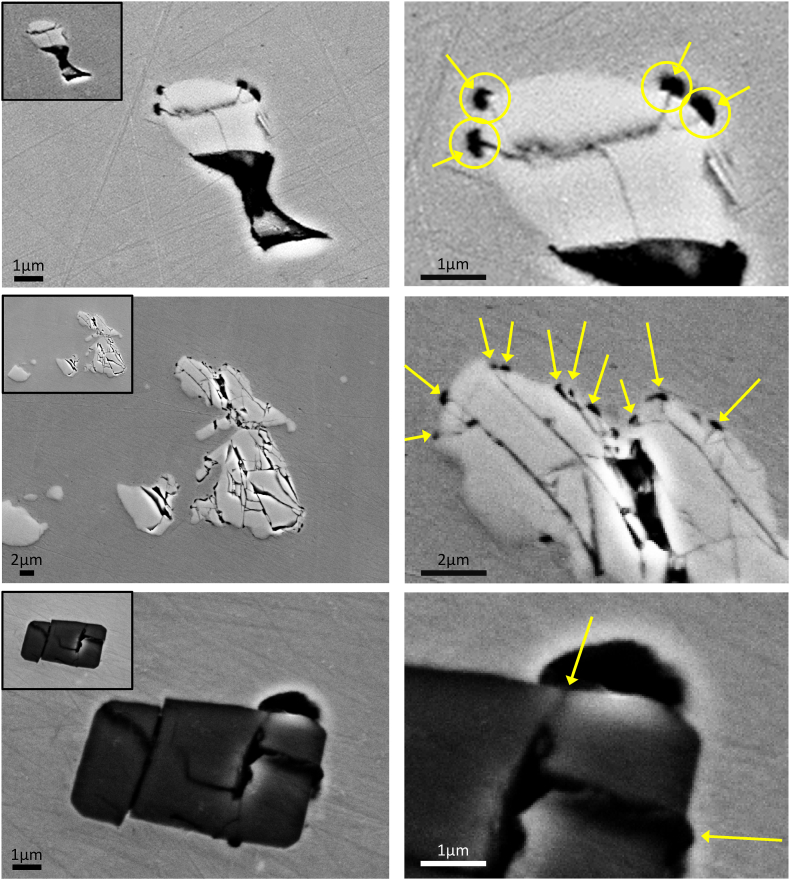
Examples of nano-cracks in particles acting as a location for corrosion initiation. The images were taken after a short period of polarization to show the first steps of localized corrosion. The detail images in the top left side show the same particles before any polarization cycle. Corrosion attacked regions are zoomed in the right side images.

Attributable to the production process, particularly the hot forming, the particles dispersed within the alloy 718 matrix endure mechanical deformation, resulting in the formation of internal nanocracks. These nanocracks are clearly visible in the images depicted in Fig. 8 and additionally presented in detail in the examples of Fig. 9. Notably, within the scope of the findings thus far, these internal nanocracks appear to be frequently associated with the initiation sites of localized corrosion events, presumably acting as a confined space (such as a crevice in crevice corrosion) that triggers corrosion initiation. As it can be seen in the evidences presented, when corrosion initiation occurs, it is always occurring in the matrix close to the particles’ nanocracks. Additionally, it was observed that grain boundaries or particles without defects (non-cracked) did not act as initiation sites for localized corrosion under the set conditions of chloride content and applied potential. When a particle presents internal nanocracks that do not finish in the boundary with the matrix, corrosion initiation related to these cracks has not been observed. An example of non-cracked particle can be seen in Fig. 9 on the top left image, where, on the left side of the corroded particle, other similar particles showing round shape and no cracks have not been attacked. These observations are supported by the research of P. Marcus et al. [25], D. E. Williams et al. [26] and M.P. Ryan et al. [27] who mention that the presence of microstructural defects on the substrate metal directly influences the quality of the protective oxide layer above those. Taking this in consideration, the presence of the matrix/particle interface, added by the nanocrack formed in the particle, that certainly creates in the matrix close to the interface a strain condition, together with the already existing void provoked by the decohesion in the interface seem to create the most vulnerable condition for corrosion initiation.

Subsequent to successive electrochemical polarizations, it has been observed that numerous cracked particles have undergone complete corrosion of the matrix in their vicinity. However, up to this point, none of these particles have been detached from the matrix.

No localized corrosion initiation has been observed on grain boundaries or within the grains without being related to the presence of the above described precipitates.

## 4 Conclusions

Based on the findings derived from the ongoing investigation outlined in this paper, we deduce that the primary μm-sized precipitates identified within alloy 718 predominantly consist of titanium nitrides, that have a dark-grey colour derived from its contrast with the matrix in the SEM imaging, and niobium carbides, which show in light-grey colour.

The devised methodology, tailored for probing the mechanisms governing the inception of localized corrosion in alloy 718, aptly serves its intended purpose. It enables the systematic investigation of the initial stages of corrosion onset in a chloride-rich environment under electrochemical polarization. Within the framework of this approach, we conclude that the application of a potential of 1050 mV vs. Ag/AgCl to alloy 718 within a 1M NaCl electrolyte results in controlled localized corrosion initiation, while forestalling the onset of general corrosion for the means of analysis.

At a potential of 1050 mV relative to Ag/AgCl in a 1 M NaCl electrolyte, corrosion initiation manifests within the matrix, proximate to its interfaces with titanium nitrides and niobium carbides, with the precipitates themselves remaining unaltered. The initiation of corrosion is correlated with pre-existing structural defects within these precipitates that act as nano-crevice-formers, creating in their surrounding a more vulnerable condition for localized corrosion initiation.

No localized corrosion initiation has been observed on grain boundaries or within the grains without being related to the presence of precipitates.

While this preliminary assessment sheds light on the initial stages of corrosion initiation taking place at the interface between the matrix and particles, where nanocracks coming from the inside of the particles are present, inquiries regarding the precise mechanisms driving this initiation process remain still open and are object of further research.

## CRediT authorship contribution statement

**Julia Botinha:** Writing – original draft, Project administration, Investigation, Formal analysis, Data curation, Conceptualization. **Bodo Gehrmann:** Writing – review & editing, Funding acquisition. **Helena Alves:** Writing – review & editing, Resources, Funding acquisition. **Michael Rohwerder:** Writing – review & editing, Supervision, Resources, Methodology, Conceptualization.

## Funding

This work was supported by the VDM Metals International GmbH.

## Declaration of competing interest

The authors declare that they have no known competing financial interests or personal relationships that could have appeared to influence the work reported in this paper.
